# Oxytocin Modulates Osteogenic Commitment in Human Adipose-Derived Stem Cells

**DOI:** 10.3390/ijms241310813

**Published:** 2023-06-28

**Authors:** Giovannamaria Petrocelli, Provvidenza Maria Abruzzo, Luca Pampanella, Riccardo Tassinari, Serena Marini, Elena Zamagni, Carlo Ventura, Federica Facchin, Silvia Canaider

**Affiliations:** 1 Department of Medical and Surgical Sciences (DIMEC), University of Bologna, Via Massarenti 9, 40138 Bologna, Italy; giovannam.petrocell2@unibo.it (G.P.); provvidenza.abruzzo2@unibo.it (P.M.A.); luca.pampanella2@unibo.it (L.P.); serena.marini2@studio.unibo.it (S.M.); e.zamagni@unibo.it (E.Z.); silvia.canaider@unibo.it (S.C.); 2 Eldor Lab, Via Corticella 183, 40129 Bologna, Italy; riccardo.tassinari.rt@gmail.com; 3 IRCCS Azienda Ospedaliero-Universitaria di Bologna, Istituto di Ematologia “Seràgnoli”, 40138 Bologna, Italy; 4 National Laboratory of Molecular Biology and Stem Cell Bioengineering of the National Institute of Biostructures and Biosystems (NIBB) c/o Eldor Lab, Via Corticella 183, 40129 Bologna, Italy

**Keywords:** human adipose-derived stem cells, oxytocin, proliferation, migration, senescence, osteogenesis, adipogenesis, autophagy

## Abstract

Human adipose-derived stem cells (hASCs) are commonly harvested in minimally invasive contexts with few ethical concerns, and exhibit self-renewal, multi-lineage differentiation, and trophic signaling that make them attractive candidates for cell therapy approaches. The identification of natural molecules that can modulate their biological properties is a challenge for many researchers. Oxytocin (OXT) is a neurohypophyseal hormone that plays a pivotal role in the regulation of mammalian behavior, and is involved in health and well-being processes. Here, we investigated the role of OXT on hASC proliferation, migratory ability, senescence, and autophagy after a treatment of 72 h; OXT did not affect hASC proliferation and migratory ability. Moreover, we observed an increase in SA-β-galactosidase activity, probably related to the promotion of the autophagic process. In addition, the effects of OXT were evaluated on the hASC differentiation ability; OXT promoted osteogenic differentiation in a dose-dependent manner, as demonstrated by Alizarin red staining and gene/protein expression analysis, while it did not affect or reduce adipogenic differentiation. We also observed an increase in the expression of autophagy marker genes at the beginning of the osteogenic process in OXT-treated hASCs, leading us to hypothesize that OXT could promote osteogenesis in hASCs by modulating the autophagic process.

## 1. Introduction

Oxytocin (OXT) was the first peptide molecule to be biochemically identified [1]. OXT is a nonapeptide containing one internal disulfide bond; OXT, synthesized by the supraoptic nucleus and by the paraventricular nucleus of the hypothalamus, is carried along the axon to the posterior pituitary and released into the blood circulation [2]. OXT can be synthesized and released by many peripheral organs, such as the heart [3], uterus [4], and thymus [5]. OXT acts through its receptor (OXTR), which belongs to the heptahelical G protein-coupled receptor family, and it is expressed in many different types of tissues, such as nervous, reproductive, and immune systems [2], as well as in many cell types as osteoblasts and adipocytes [6,7,8,9]. 

OXT has many properties and functions related to a wide range of physiological fields, like lactation and parturition [10], energy metabolism [11], immune system [12,13], or thermoregulation and body composition [14,15]. All of these, and many other functions, influence health and well-being. 

Among the biological effects of OXT, we focused on the role of OXT on the properties and fate of human adipose-derived stem cells (hASCs), that like the other mesenchymal stem cells (MSCs), are extremely attractive candidates for cell therapy approaches [16,17]; moreover, hASCs have minimal ethical concerns and are considered to be safer than embryonic stem cells (ESCs) or induced pluripotent stem cells (iPSCs) in terms of tumorigenesis or genomic modifications [18].

To date, several studies investigated the role of OXT in hMSCs, and data revealed interesting disclosures. OXT protects rat bone marrow-derived MSCs (rat BM-MSCs) against the cytotoxic and apoptotic effects of hypoxia and serum deprivation [19]. Kim and collaborators demonstrated a positive effect of OXT on the migration ability of MSCs isolated from human umbilical cord blood (hUCB-MSCs) [20]. It is also known that OXT promotes tissue repair and cell differentiation of stem cells [6]. Many types of research have addressed the role of OXT on cardiogenesis, which is mediated by OXTR [21]: OXT stimulates cardiac differentiation both in P19 cells [21] and in embryoid bodies obtained from mouse ESCs [22] as well as in adult murine Sca-1-positive cells that, in the adult hearts, have some characteristics of stem cells [23]; the same cardiac differentiation effect was observed in porcine BM-MSCs [24], in mouse ASCs [25], in UCB-MSCs [26], and in iPSC-derived epicardial cells [27]. OXT also stimulates the stem cell chondrogenic differentiation [28], making this molecule an interesting candidate for osteoarthritis treatment [29]: in fact, in hBM-MSCs and hASCs, OXT enhances the mRNA expression of genes involved in chondrogenesis [28]. OXT also improves muscle regeneration by enhancing aged muscle stem cell activation and proliferation through the Mitogen-Activated Protein Kinase (MAPK)/Extracellular Signal-Regulated Kinase (ERK) signaling pathway [30], as well as liver regeneration, especially in aged mice [6]. Recent studies have proposed the involvement of OXT also in neurogenesis, since OXT increased the expression of neural-specific markers in hASCs, probably due to an enhancement of Ca^2+^ release from intracellular stores that blocks the Bone Morphogenetic Protein 4 (BMP4), a neurogenesis inhibitor [31]. 

OXT is also implicated in bone tissue homeostasis; in fact, functional disequilibria in OXT was observed in bone-related pathologies like osteoporosis [32]. Osteoporosis leads to a loss of bone mass and an increase in adipose tissue, thus suggesting the existence of a physiological balance between bone tissue and adipose tissue [33]. Studies performed on hASCs and hBM-MSCs have shown that OXT is able to stimulate osteogenic differentiation by inhibiting adipogenesis, highlighting the correlation between osteoporosis and reduced OXT levels in mice plasma, and demonstrating that the administration of OXT in ovariectomized mice induces osteogenic differentiation and allows the recovery of the biomechanical properties of bone [32]. OXT-stimulated osteogenic differentiation was also observed in human periodontal ligament-derived stem cells (hPDLSCs) [34], which represent an ideal source of cells for the repair and regeneration of lost periodontal tissue [35]. Moreover, OXT promoted hPDLSC proliferation and migration [34]. 

However, to our knowledge, the effect of OXT on the ability of hASCs to differentiate in osteocytes or adipocytes is poorly investigated. Thus, the aim of this study was to analyze the role of OXT in hASC osteogenesis and adipogenesis, as well as to provide a comprehensive framework of the effects of OXT on other biological properties, such as proliferation, senescence, and migration. 

## 2. Results

### 2.1. Characterization of hASCs

The hASCs were isolated and characterized as previously described [36]. They expressed the major surface markers of MSCs (CD73, CD90, CD105), whereas they did not show the hematopoietic related surface markers (CD34 and CD45), showed the typical fibroblast-like shape of MSCs and were able to differentiate toward adipogenic, osteogenic (also demonstrated here in Section 2.7.1 and Section 2.7.2, respectively), and chondrogenic lineages when they were cultured with appropriate differentiation media [36].

### 2.2. Effects of Oxytocin on hASC Morphology and Oxytocin Receptor mRNA Expression

**Figure 1:**
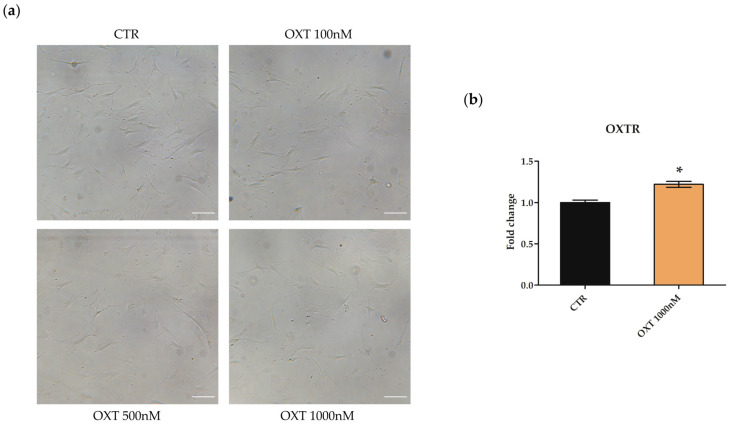
Human adipose-derived stem cell (hASC) morphology and Oxytocin receptor (OXTR) mRNA expression. (**a**) Representative images of hASCs morphology after 72 h of treatment with different concentrations of Oxytocin (OXT). hASCs were seeded at 3500 cells/cm^2^ and treated with OXT at 100, 500, and 1000 nM; untreated cells were used as control (CTR). Cells were detected under bright field illumination using a Leica Labovert FS Inverted Microscope and Leica MC170 HD Imaging System Camera. Scale bars: 100 μm. (**b**) *OXTR* mRNA expression in CTR and OXT-treated hASCs. The mRNA expression of *OXTR* was assessed in hASCs after 72 h of basal culture condition (CTR) or after 72 h of OXT 1000 nM treatment. Data obtained by quantitative Real-Time PCR (qPCR) were normalized using three reference genes (*TATA Binding Protein*—*TBP*, *Glyceraldehyde-3-Phosphate Dehydrogenase*—*GAPDH* and *Hypoxanthine Phosphoribosyltransferase 1*—*HPRT1*); the normalized expression value of CTR cells was set to 1, and *OXTR* gene expression value of OXT-treated cells was reported to CTR value. The graphic represents the normalized fold change ± standard error of the mean (SEM); *n* = 3; * *p* < 0.05.

hASCs were treated with different concentrations of OXT (100, 500, and 1000 nM) for 72 h. As shown in Figure 1a, none of the OXT concentrations used influenced hASC morphology compared to untreated (CTR) hASCs (Figure 1a). To evaluate whether hASCs expressed the OXTR and whether OXT modulated its expression, the *OXTR* mRNA abundance was assessed by quantitative Real-Time PCR (qPCR) analysis on both CTR and hASCs treated with 1000 nM OXT. hASCs expressed the *OXTR* gene, and OXT treatment significantly increased its expression (Figure 1b).

### 2.3. Effects of Oxytocin on hASC Viability

To evaluate the effects of different OXT concentrations (100, 500, and 1000 nM) on hASC viability, cells were counted 72 h after OXT treatment in the presence of the Erythrosine B dye that allows discriminating between dead (red) and living cells (unstained). The percentage of living cells for both CTR and OXT-treated cells was comparable, indicating that OXT was not toxic for hASCs at the tested concentrations (Table 1). 

### 2.4. Effects of Oxytocin on hASC Proliferation

**Figure 2:**
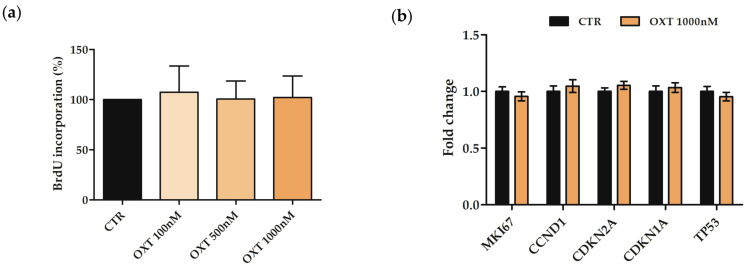
Proliferative ability of human adipose-derived stem cells (hASCs) after 72 h of treatment with Oxytocin (OXT). (**a**) Bromodeoxyuridine (BrdU) assay was used to assess cell proliferation in untreated (CTR) or treated cells with OXT (100, 500, and 1000 nM) for 72 h. Histograms represent the mean percentage of BrdU incorporation normalized to CTR ± standard deviation (SD); *n* = 3. (**b**) Expression of cell cycle and proliferation marker genes in CTR and in 1000 nM OXT-treated hASCs. The expression of *marker of proliferation Ki-67* (*MKI67*), *cyclin D1* (*CCND1*), *cyclin-dependent kinase inhibitor 2A* (*CDKN2A*, alias *p16^INK4a^*), *cyclin-dependent kinase inhibitor 1A* (*CDKN1A*, alias *p21*) and *tumor protein p53* (*TP53*) genes was assessed by quantitative Real-Time PCR (qPCR) after 72 h of OXT treatment. Data were normalized using four reference genes (*Ribosomal Protein L13a*—*RPL13a*, *TATA Binding Protein*—*TBP*, *Glyceraldehyde-3-Phosphate Dehydrogenase*—*GAPDH*, *Hypoxanthine Phosphoribosyltransferase 1—HPRT1*); for each gene, the normalized expression value of CTR was set up to 1, and the gene expression value of the OXT treated sample was reported to CTR value. Data are reported as normalized fold change ± standard error of the mean (SEM); *n* = 3.

Since the cell count showed comparable viability between hASCs treated with OXT and CTR cells, to evaluate the effects of OXT on hASC proliferation, a Bromodeoxyuridine (BrdU) assay was performed. hASC samples (CTR and treated cells with OXT at 100, 500, and 1000 nM for 72 h) were incubated with a BrdU labeling solution. The resulting amount of positive BrdU cells in CTR was comparable with the amount in OXT-treated cells, and no significant differences were revealed between samples (Figure 2a). To confirm proliferation results, the expression of genes involved in cell proliferation and cell cycle control was investigated in both CTR and treated hASCs only with 1000 nM OXT for 72 h. The investigated genes were *markers of proliferation Ki-67* (*MKI67*), *cyclin D1* (*CCND1*), *cyclin-dependent kinase inhibitor 2A* (*CDKN2A*, alias *p16^INK4a^*), *cyclin-dependent kinase inhibitor 1A* (*CDKN1A*, alias *p21*) and *tumor protein p53* (*TP53*). Data obtained did not show any significant differences in the expression of all investigated genes between CTR and OXT-treated hASCs, thus confirming that OXT did not affect hASC proliferation when used at this concentration (Figure 2b).

### 2.5. Effects of Oxytocin on hASC Migration Ability

**Figure 3:**
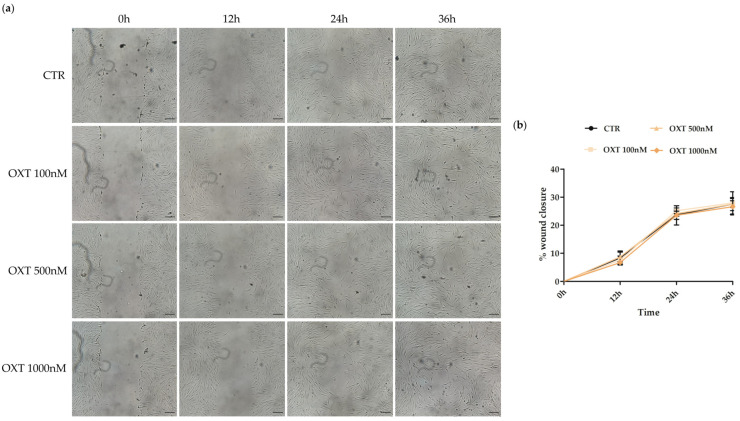
Scratch wound healing assay in human adipose-derived stem cells (hASCs) treated or untreated with Oxytocin (OXT). The migratory ability of hASCs to close the wound in untreated (CTR) and in OXT (100, 500, and 1000 nM) treated cells were analyzed after a treatment of 24 h. (**a**) Images were acquired immediately after scratch (0 h) and at the indicated time-points (12, 24, 36 h) using the Leica Labovert FS Inverted Microscope equipped with a Leica MC170 HD Imaging System Camera. Scale bars: 100 μm. (**b**) Evaluation of the percentage of wound closure at different time points (0, 12, 24, 36 h) in CTR and OXT-treated cells. The scratch area was measured using the ImageJ software 1.53e. The results are presented as the mean percentage of wound closure normalized on the CTR cells ± standard error of the mean (SEM). The percentage of wound closure at t = 0 h was set to 0 for both CTR and OXT-treated cells; *n* = 3.

To assess the effect of OXT on another biological property of hASCs, we investigated the role of OXT on their migratory capacity. Scratch wound healing assay was performed in hASCs after treating them with or without OXT at 100, 500, and 1000 nM for 24 h. As shown in Figure 3, cells treated with OXT showed no difference in the ability to close the scratch “wound” compared to CTR cells for all the investigated conditions (Figure 3); these findings were consistent with the proliferation data reported above.

### 2.6. Effects of Oxytocin on hASC Senescence-Associated-β-Galactosidase (SA-β-Gal) Activity and Autophagy

**Figure 4:**
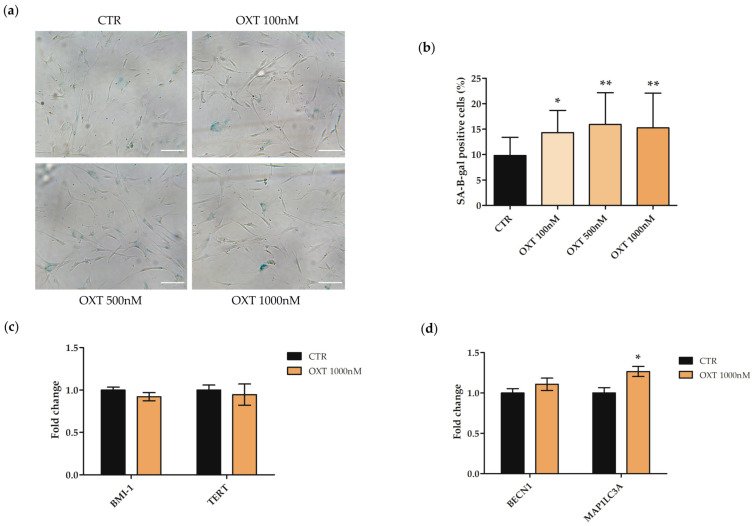
Evaluation of cell senescence and analysis of the expression of senescence/autophagy marker genes in human adipose-derived stem cells (hASCs) treated with Oxytocin (OXT). (**a**) Representative images of Senescence-Associated-β-Galactosidase (SA-β-Gal) staining assay in hASCs untreated (CTR) or treated with OXT (100, 500, and 1000 nM) for 72 h. Positive cells are blue and negative cells are unstained. Scale bars = 100 μm. (**b**) The graph represents the percentage of SA-β-Gal positive cells ± standard deviation (SD); cells were counted in five random fields for each technical replicate under the light microscope Leica Labovert FS Inverted Microscope equipped with a Leica MC170 HD Imaging System Camera; *n* = 3; * *p* < 0.05, ** *p* < 0.01 vs. CTR. (**c**) Expression of *BMI1 proto-oncogene, polycomb ring finger* (*BMI-1*) and *telomerase reverse transcriptase (TERT*) genes in hASCs CTR or treated with 1000 nM OXT for 72 h. Data obtained by quantitative Real-Time PCR (qPCR) were normalized using three reference genes (*TATA Binding Protein*—*TBP*, *Glyceraldehyde-3-Phosphate Dehydrogenase—GAPDH,* and *Hypoxanthine Phosphoribosyltransferase 1*—*HPRT1*). (**d**) Expression of *Beclin 1* (*BECN1*) and *microtubule-associated protein 1 light chain 3 alpha* (*MAP1LC3A*) genes in hASCs CTR or treated with 1000 nM OXT for 72 h. Data obtained by qPCR were normalized using three reference genes (*TBP*, *GAPDH*, and *HPRT1*). In (**c**,**d**), in each graph and for each gene, the normalized expression values of CTR cells were set up to 1, and all other gene expression values were reported to that sample. Data are reported as normalized fold change ± standard error of the mean (SEM); *n* = 3; * *p* < 0.05.

To evaluate whether OXT could modulate hASC senescence, the SA-β-Gal staining assay was performed in untreated (CTR) or in treated hASCs with OXT (100, 500, and 1000 nM) for 72 h. The number of positively blue-stained cells, calculated as the percentage of total number of cells, was significantly higher in the treated cells compared to CTR at each OXT concentration investigated (Figure 4a,b). Therefore, we investigated the expression of marker genes involved in cell senescence *telomerase reverse transcriptase* (*TERT*) and *BMI1 proto-oncogene, polycomb ring finger* (*BMI-1*) in hASCs treated for 72 h with OXT at 1000 nM, the dose in which the most evident effect on SA-β-Gal activity was observed. Data reported in Figure 4c showed that OXT did not affect the expression of both *TERT* and *BMI-1*. The increase in SA-β-Gal activity is not only associated with cellular senescence, but may be related to other cellular pathways in which the lysosomal activity is involved, such as autophagy [37,38]. Thus, the expression levels of two autophagic marker genes, *Beclin 1* (*BECN1*) and *microtubule-associated protein 1 light chain 3 alpha (MAP1LC3A*), were investigated by qPCR in untreated or 1000 nM OXT-treated hASCs for 72 h. Data shown in Figure 4d indicated that 1000 nM OXT induced an increase in the gene expression of *BECN1* and, significantly, of *MAP1LC3A* in hASCs, compared to CTR, indicating a possible involvement of OXT in the autophagy pathway (Figure 4d). 

### 2.7. Effects of Oxytocin on the Adipogenic and Osteogenic Potential of hASCs

**Figure 5:**
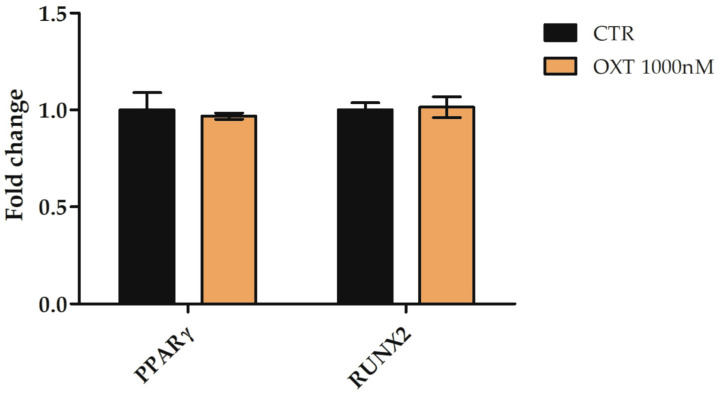
Expression of *peroxisome proliferator-activated receptor gamma* (*PPARγ*) and *RUNX family transcription factor 2* (*RUNX2*) genes in human adipose-derived stem cells (hASCs) treated or untreated with Oxytocin (OXT) for 72 h. hASCs were cultured without (CTR) or with 1000 nM OXT for 72 h, and the expression of *PPARγ* and *RUNX2* genes were assessed. Data obtained by quantitative Real-Time PCR (qPCR) were normalized using three reference genes (*TATA Binding Protein*—*TBP*, *Glyceraldehyde-3-Phosphate Dehydrogenase*—*GAPDH*, and *Hypoxanthine Phosphoribosyltransferase 1*—*HPRT1*). For each gene, the normalized expression value of CTR was set up to 1, and other gene expression values were reported to that sample. Data were reported as normalized fold change ± standard error of the mean (SEM); *n* = 3.

The effects of OXT on hASC differentiation ability were also evaluated; in particular, we focused on adipogenic and osteogenic commitment. The expression of *peroxisome proliferator-activated receptor gamma* (*PPARγ)* and *RUNX family transcription factor 2* (*RUNX2*) genes, two important adipogenic and osteogenic markers, respectively, was analyzed in hASCs after only 72 h of OXT treatment to evaluate whether OXT alone could address hASCs towards a specific commitment, in the absence of specific induction media. The expression of both *PPARγ* and *RUNX2* genes was not altered in hASCs treated with OXT 1000 nM compared to CTR cells, suggesting that this peptide alone was not able to induce adipogenic and osteogenic differentiation (Figure 5). 

#### 2.7.1. Effects of Oxytocin on the hASC Adipogenic Commitment

The effects of OXT on hASC adipogenic commitment were analyzed by culturing cells with OXT in the adipogenic induction medium for the entire period of the differentiation protocol. Adipogenesis was monitored by counting the number of the Oil Red O (O.R.O) positive cells, as well as by evaluating the expression of the adipogenic gene marker *PPARγ*.

**Figure 6:**
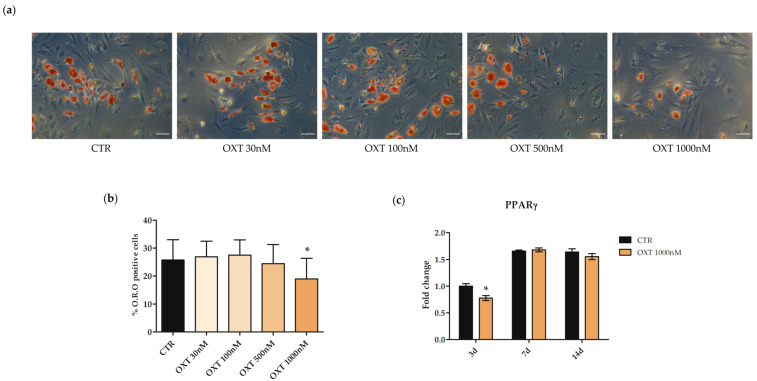
Adipogenic potential of human adipose-derived stem cells (hASCs) in the presence of Oxytocin (OXT). hASC adipogenic differentiation was induced by culturing cells in the StemPro^®^ Adipogenesis Differentiation medium without (CTR) or with OXT at 30, 100, 500, or 1000 nM for 14 days. The final adipogenic commitment was evaluated by Oil Red O (O.R.O) staining. (**a**) Representative O.R.O images were acquired under phase contrast with the microscope Leica Labovert FS Inverted Microscope equipped with the Leica MC170 HD Imaging System. Cells positive for adipogenesis showed red vacuoles in the cytoplasm. Scale bars: 50 μm. (**b**) The graph shows data obtained by counting undifferentiated unstained and red O.R.O-stained cells using ImageJ software 1.53e. Data are reported as the mean percentage of O.R.O positive cells ± standard deviations (SD); *n* = 3; * *p* < 0.05 vs. CTR. (**c**) Expression of the adipogenic marker gene *peroxisome proliferator-activated receptor gamma* (*PPARγ*) in hASCs CTR or treated with OXT 1000 nM for the entire adipogenic protocol. The expression of the *PPARγ* gene was assessed at 3, 7, and 14 days (d) from the beginning of the protocol. Data obtained by quantitative Real-Time PCR (qPCR) were normalized using three reference genes (*TATA Binding Protein*—*TBP*, *Glyceraldehyde-3-Phosphate Dehydrogenase*—*GAPDH*, *Hypoxanthine Phosphoribosyltransferase 1*—*HPRT1*); the normalized expression value of CTR at 3 days (black column 3d) was set up to 1, and all other gene expression values were reported to that sample. Data were reported as normalized fold change ± standard error of the mean (SEM); *n* = 3; * *p* < 0.05 vs. CTR 3d.

hASCs were treated for the whole duration of the adipogenic protocol with OXT 30, 100, 500, and 1000 nM; after 14 days, a significant reduction in adipogenic commitment was observed in hASCs cultured in adipogenic differentiation medium and treated with 1000 nM OXT, compared to CTR cells (Figure 6a,b). Moreover, *PPARγ* gene expression was investigated in CTR and in 1000 nM OXT-treated hASCs at 3, 7, and 14 days from the beginning of the adipogenic differentiation protocol. Consistent with O.R.O staining results, *PPARγ* gene expression was statistically reduced in OXT-treated cells compared to CTR cells after 3 days from the beginning of adipogenesis induction, consistent with the knowledge that *PPARγ* is an early adipogenic marker gene (Figure 6c). 

#### 2.7.2. Effects of Oxytocin on the hASC Osteogenic Commitment

The effects of OXT on hASC osteogenic commitment were analyzed by culturing cells with OXT in the induction medium for the whole duration of the differentiation protocol. Osteogenesis was monitored by evaluating the amount of the Alizarin Red S dye, the mRNA expression of the osteogenic gene marker *bone gamma-carboxyglutamate protein* (*BGLAP*, also named osteocalcin) as well as the protein expression of the RUNX2, osteopontin (OPN), type 1 collagen and fibronectin.

**Figure 7:**
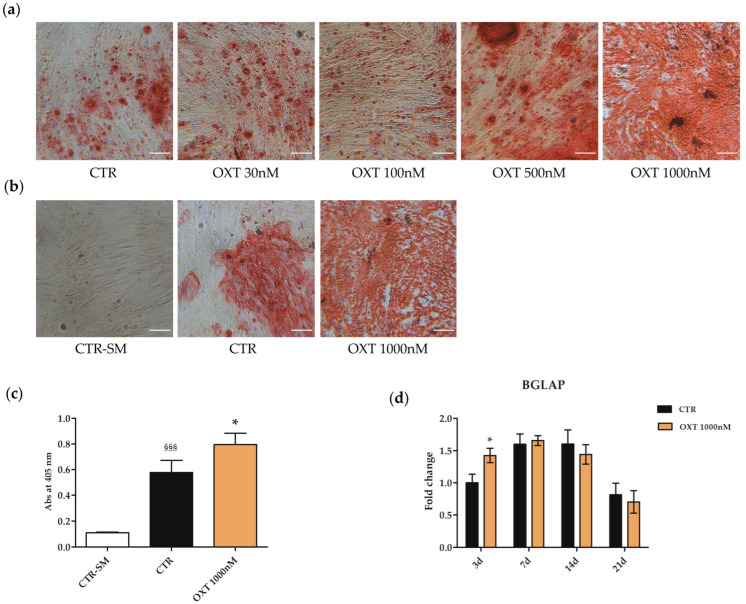
Osteogenic potential of human adipose-derived stem cells (hASCs) treated with Oxytocin (OXT). hASCs were induced to differentiate into the osteogenic lineage for a total period of 21 days. Cells were cultured in a standard medium (SM) or in an osteogenic differentiation medium (OM) without (CTR) or OXT. The final osteogenic commitment was evaluated by Alizarin Red S staining. (**a**) Representative images of hASCs cultured in OM and untreated (CTR) or treated with OXT at four different concentrations (30, 100, 500, and 1000 nM). (**b**) Representative images of three independent experiments where hASCs were cultured in SM without OXT (CTR-SM) or in OM without (CTR) or with OXT 1000 nM. Images (**a**,**b**) were acquired using the microscope Leica Labovert FS Inverted Microscope equipped with Leica MC170 HD Imaging System Camera. The presence of the red-colored calcium deposits indicated the osteogenesis process. Scale bars: 100 μm. (**c**) Alizarin Red S dye incorporated into the matrix was solubilized with 10% acetic acid, and absorbance (Abs) was read at 405 nm with a spectrophotometer plate reader (Wallac 1420 Victor2 Multilabel Counter). Data were expressed as mean of Alizarin Red S Abs at 405 nm ± standard deviation (SD); *n* = 3; * *p* < 0.05 vs. CTR; ^§§§^ *p* < 0.001 vs. CTR-SM. (**d**) Expression of *bone gamma-carbossyglutamic acid-containing protein* (*BGLAP*) gene in hASCs cultured in OM without or with OXT 1000 nM, assessed at 3, 7, 14 and 21 days (d) from the beginning of the osteogenic protocol. Data obtained by quantitative Real-Time PCR (qPCR) assay were normalized using three reference genes (*TATA Binding Protein*—*TBP*, *Glyceraldehyde-3-Phosphate Dehydrogenase*—*GAPDH*, *Hypoxanthine Phosphoribosyltransferase 1*—*HPRT1*); the normalized expression value of CTR at 3 days (black column 3d) was set up to 1, and all other gene expression values were reported to that sample. Data were reported as normalized fold change ± standard error of the mean (SEM); *n* = 3; * *p* < 0.05 vs. CTR 3d.

In the first experiment, four OXT concentrations (30, 100, 500, and 1000 nM) were used during the entire osteogenic protocol. The Alizarin Red S staining, which specifically binds calcium deposits, revealed a dose-dependent increase in the osteogenic commitment in cells cultured in an osteogenic medium (OM) and treated with OXT, especially at the concentration of 1000 nM (Figure 7a). Thus, three more independent experiments were performed using only the 1000 nM OXT concentration. Images and Alizarin Red S staining quantification data showed that OXT treatment induced an increase in the osteogenic differentiation potential of hASCs cultured in OM compared to the control counterpart (hASCs cultured in osteogenic medium without OXT treatment) (Figure 7b,c). Osteogenesis was then evaluated by measuring the mRNA abundance of the *BGLAP* gene in hASCs cultured in OM and treated without or with 1000 nM OXT for 3, 7, 14, and 21 days from the beginning of the osteogenic induction protocol. The expression of *BGLAP* followed the same expression pattern in both CTR and OXT-treated cells, i.e., an increase and then a decrease in *BGLAP* expression over time, but in OXT-treated hASCs an increase in *BGLAP* expression was observed after 3 days from the beginning of the protocol compared to CTR (Figure 7d), suggesting that the OXT treated cells were prone to initiate osteogenesis earlier than the untreated ones. 

**Figure 8:**
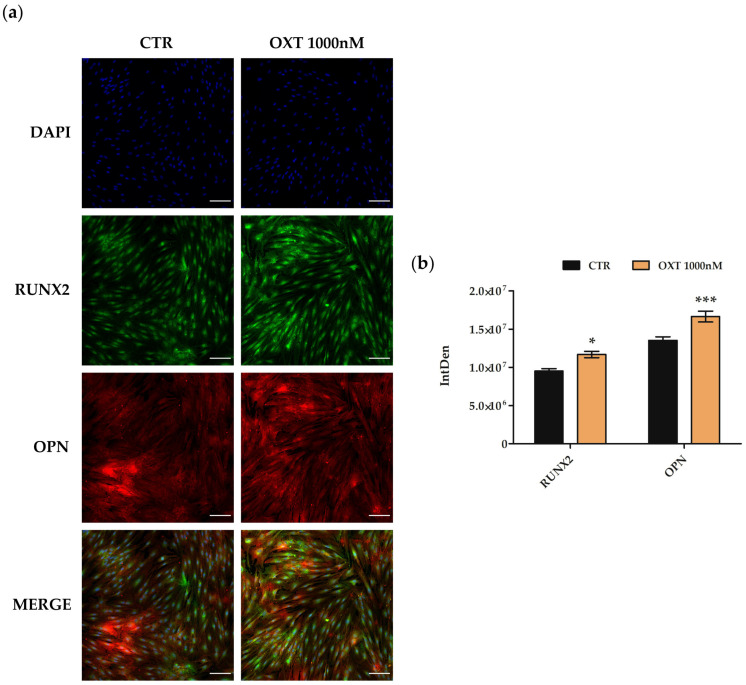
Immunofluorescence of osteogenic markers in human adipose-derived stem cells (hASCs) after Oxytocin (OXT) treatment during the osteogenic protocol. hASCs treated or not (CTR) with 1000 nM OXT were immunostained with NucBlue^®^ Fixed Cell Ready Probes^®^ Reagent (DAPI, blu signal, specific to counterstain nuclei), with anti-RUNX family transcription factor 2 (RUNX2, green signal) and anti-osteopontin (OPN, red signal) antibodies. (**a**) Representative images of CTR and 1000 nM OXT-treated hASCs after 3 days of the osteogenic protocol. Images were acquired using the Nikon inverted microscope Eclipse Ti2-E (Nikon Instruments, Melville, NY, USA) and a digital sight camera DS-Qi2 (Nikon Instruments, Melville, NY, USA) through the imaging software NIS-Elements. Scale bars: 100 μm (**b**) Fluorescence intensity of CTR and 1000 nM OXT-treated hASCs samples after 3 days of the osteogenic protocol. Fluorescence was measured by Fiji software 2.1.0/1.53c, and it is reported as IntDen (Integrated Density), referring to the average value of fluorescence in a selected area normalized to the number of cells. Data are expressed as the mean IntDen ± standard error of the mean (SEM); *n* = 3; * *p* < 0.05 and *** *p* < 0.001.

Finally, osteogenesis was investigated by analyzing the expression of specific osteogenic proteins by immunofluorescence. First, protein expression of RUNX2 and OPN was evaluated in hASCs cultured in an osteogenic medium with or without 1000 nM OXT after 3, 7, 14, and 21 days from the beginning of the osteogenic protocol. Images and fluorescence quantifications showed that both RUNX2 and OPN were statistically more expressed in OXT-treated hASCs compared to CTR cells after 3 days from the start of osteogenic protocol (Figure 8), while they are comparable at 7, 14, and 21 days.

**Figure 9:**
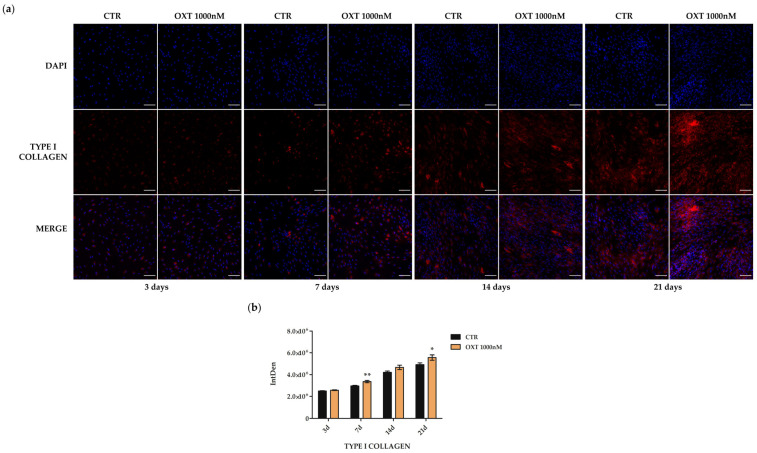
Immunofluorescence of type 1 collagen in human adipose-derived stem cells (hASCs) treated with Oxytocin (OXT) during the osteogenic protocol. Untreated (CTR) and OXT 1000 nM treated hASCs were immunostained with NucBlue^®^ Fixed Cell Ready Probes^®^ Reagent (DAPI, blu signal, specific to counterstain nuclei) and anti-type 1 collagen (red signal) antibody. (**a**) Representative images of CTR hASCs and hASCs treated with OXT 1000 nM after 3, 7, 14, and 21 days of the osteogenic differentiation protocol. Images were acquired using the Nikon inverted microscope Eclipse Ti2-E (Nikon Instruments, Melville, NY, USA) and a digital sight camera DS-Qi2 (Nikon Instruments, Melville, NY, USA) through the imaging software NIS-Elements. Scale bars: 100 μm. (**b**) Fluorescence intensity of CTR and 1000 nM OXT-treated hASCs samples after 3, 7, 14, and 21 days (d) of the osteogenic protocol. Fluorescence was measured by Fiji software 2.1.0/1.53c, and it is reported as IntDen (Integrated Density), referring to the average value of fluorescence in a selected area normalized to the number of cells. Data are expressed as the mean IntDen ± standard error of the mean (SEM); *n* = 3; * *p* < 0.05 and ** *p* < 0.01.

Immunofluorescence staining images and the relative fluorescence quantification revealed that the expression of type I collagen was evident from the third day of the osteogenic protocol and that both CTR and OXT-treated hASCs showed both intracellular and extracellular collagen at 14 and 21 days (Figure 9). Type I collagen increased in OXT-treated cells compared to untreated cells at days 7, 14, and 21 of the osteogenic process, reaching statistical significance both at 7 and 21 days (Figure 9). 

**Figure 10:**
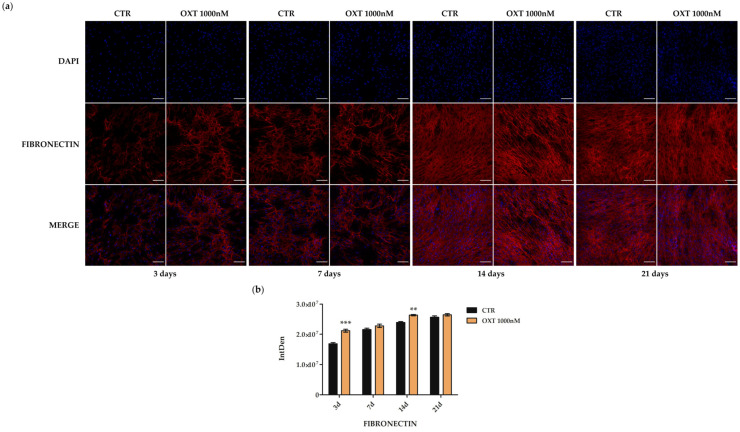
Immunofluorescence of fibronectin in human adipose-derived stem cells (hASCs) after Oxytocin (OXT) treatment during the osteogenic protocol. Untreated (CTR) and OXT 1000 nM treated hASCs were immunostained with NucBlue^®^ Fixed Cell Ready Probes^®^ Reagent (DAPI, blu signal, specific to counterstain nuclei) and anti-fibronectin (red signal) antibody. (**a**) Representative images of CTR and OXT 1000 nM treated hASCs after 3, 7, 14, and 21 days of the osteogenic differentiation protocol. Images were acquired using the Nikon inverted microscope Eclipse Ti2-E (Nikon Instruments, Melville, NY, USA) and a digital sight camera DS-Qi2 (Nikon Instruments, Melville, NY, USA) through the imaging software NIS-Elements. Scale bars: 100 μm. (**b**) Fluorescence intensity of CTR and 1000 nM OXT-treated hASCs samples after 3, 7, 14, and 21 days (d) of the osteogenic protocol. Fluorescence was measured by Fiji software 2.1.0/1.53c, and it is reported as IntDen (Integrated Density), referring to the average value of fluorescence in a selected area normalized to the number of cells. Data are expressed as the mean IntDen ± standard error of the mean (SEM); *n* = 3; ** *p* < 0.01 and *** *p* < 0.001.

Finally, fibronectin was more expressed in OXT-treated cells than in untreated cells from day 3 of the induction protocol for the entire period analyzed, significantly at days 3 and 14 (Figure 10).

**Figure 11:**
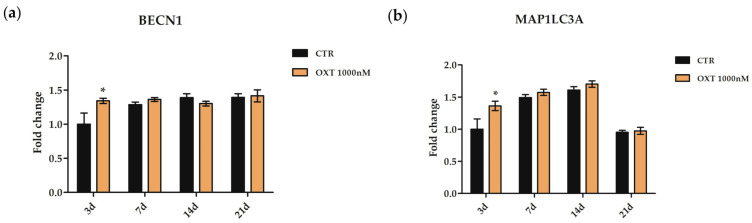
Gene expression of autophagic markers along the osteogenic program in human adipose-derived stem cells (hASCs) treated with Oxytocin (OXT). hASCs were cultured with or without (CTR) OXT 1000 nM. (**a**) Expression of *Beclin 1* (*BECN1*) and (**b**) *microtubule-associated protein 1 light chain 3 alpha* (*MAP1LC3A*) genes in hASCs CTR or treated with 1000 nM OXT was analyzed after 3, 7, 14, and 21 days (d) from the beginning of the osteogenic differentiation protocol. Data obtained with quantitative Real-Time PCR (qPCR) were normalized using three reference genes (*TATA Binding Protein*—*TBP*, *Glyceraldehyde-3-Phosphate Dehydrogenase*—*GAPDH,* and *Hypoxanthine Phosphoribosyltransferase 1*—*HPRT1*). In each graph, the normalized expression value of CTR hASCs at 3 days (black column 3d) was set up to 1, and all other gene expression values were reported to that sample. Data are reported as normalized fold change ± standard error of the mean (SEM); *n* = 3; * *p* < 0.05 vs. CTR 3d.

Finally, since OXT modulated autophagy in hASCs after 72 h of treatment, we evaluated the expression of two autophagy marker genes (*BECN1* and *MAP1LC3A*) along the entire osteogenic process. Cells were cultured in the osteogenic medium as previously described and treated with or without 1000 nM OXT. For each condition, RNA was extracted 3, 7, 14, and 21 days after the start of treatment. Both genes significantly increased their expression in OXT-treated cells on day 3 of the induction protocol compared to CTR cells, while they showed the same expression pattern on days 7, 14, and 21 (Figure 11).

## 3. Discussion

The aim of this study was to understand whether OXT could affect the main biological proprieties of hASCs, such as morphology, proliferation, viability, and senescence, as well as their differentiation potential. hASCs, like other MSCs, show peculiar features that make them extremely attractive actors in the regenerative physiological processes and intriguing candidates for cell therapy approaches in the wider field of regenerative medicine.

Our data demonstrated that OXT did not influence hASC morphology at all OXT concentrations used and enhanced the expression of OXTR, which was expressed even at the basal level in hASCs. Our data are consistent with previously published data showing that OXTR was expressed in adipocytes [7] as well as in mouse ASCs [31] or hASCs [32]. Our observation that OXT enhanced the transcription of its own receptor coding gene suggests that OXT itself may be engaged in tonic feedforward mechanisms sustaining its action in an autocrine/paracrine fashion.

Moreover, under our experimental conditions, OXT did not affect cell viability, as well as cell proliferation, both at cellular and molecular levels. As a matter of fact, the expression of the genes involved in cell proliferation and cell cycle control *MKI67*, *CCND1*, *CDKN2A*, *CDKN1A,* and *TP53* was not affected in OXT-treated cells, confirming that OXT did not influence cell proliferation. However, other researchers observed that OXT improved MSC proliferation, particularly in hPDLSCs and rat BM-MSCs [19,34]. This discrepancy could be related to the different cellular models as well as to different OXT concentrations which were used.

A coherent result was obtained from the migration assay where cells treated with OXT for 24 h showed no differences in the ability to close the scratch “wound” compared to CTR cells, even here in contrast with other studies where OXT promoted migration, although in different MSC models, as hUCB-MSCs, hPDLSCs, and rat BM-MSCs [20,34].

We then investigated the role of OXT in hASC senescence. Surprisingly, we found an increase in the number of positively blue-stained cells in hASCs treated with OXT. Since the induction of cellular senescence appeared to be in contrast with the general role of OXT mainly involved in health processes, we investigated the senescence pathway by analyzing the expression of cell senescence genes such as *TERT* and *BMI-1*: OXT did not alter the expression of both genes, suggesting that the increase on SA-β-Gal activity could not be related to the induction of a senescence process. In fact, it is known that the increase in SA-β-Gal activity is associated with other cellular processes, such as autophagy, in which an increase in lysosomal activity is required [37,38]. Therefore, we investigated the expression of two autophagic marker genes, *BECN1* and *MAP1LC3A*, encoding proteins implicated in the autophagosome formation during the autophagy process [39,40]. The increase in the expression of both genes in hASCs treated with OXT indicated a possible involvement of OXT in the autophagy process, as already described in the mouse hepatocytes cell line AML12 [6], but never demonstrated in MSC models.

Finally, we analyzed the role of OXT on hASC differentiation ability, particularly focusing on both adipogenesis and osteogenesis. OXT was added to the adipogenic or osteogenic induction medium for the entire period of the differentiation protocol.

As demonstrated both by O.R.O staining and by the evaluation of *PPARγ* gene expression, lower concentrations of OXT did not influence the adipogenic process, which was instead reduced by the OXT’s highest dose. These data confirmed the inclination of OXT to reduce the adipogenic potential already described by another research, but with a much lower dose of OXT [32].

On the other hand, osteogenesis was clearly promoted by OXT in a dose-dependent manner, and the most evident result was obtained with OXT at 1000 nM. Therefore, we studied the modulation of osteogenesis deeply throughout its process when OXT at 1000 nM was used on hASCs. The increase in red staining of Alizarin in OXT-treated cells was confirmed by the significant increase in *BGLAP* gene expression three days after the start of the osteogenic protocol; the pattern of *BGLAP* expression was similar in CTR and OXT-treated hASCs during the osteogenic protocol, but the evident increase in its expression at 3 days could suggest that OXT stimulated the cells to the osteogenic process, acting in the first phase of the differentiation, leading to an early expression of osteogenic markers compared to CTR. Akin to this view, two osteogenic marker proteins, RUNX2 and OPN, were statistically more expressed in OXT-treated hASCs than in untreated cells 3 days after initiation of the osteogenic protocol, strengthening the hypothesis of a push by OXT on hASCs towards osteogenic commitment in the first step of the process. 

Autophagy is known to play a key role in maintaining stemness properties and modulating stem cell differentiation [41,42], including osteogenic differentiation of human MSCs [43,44]. Since OXT alone was able to induce the expression of *BECN1* and *MAP1LC3A*, we hypothesized that the potentiation of osteogenic differentiation in OXT-treated hASCs may be due to OXT’s ability to modulate the autophagy process. The data seem to support our hypothesis; in fact, the gene expression of *BECN1* and *MAP1LC3A* increased significantly in OXT-treated hASCs at day 3 of osteogenic induction, as occurred with the osteogenic marker genes and proteins studied. This evidence suggests that OXT could promote osteogenesis through the potentiation of the autophagic process. Moreover, it is known that autophagy is closely linked to lysosomal activity, which is enhanced during osteogenic differentiation [45]. Thus, the increase in SA-β-Gal activity in OXT-treated cells might reinforce the hypothesis that OXT-induced osteogenic differentiation regulating autophagy and the lysosomal function.

In addition, type 1 collagen and fibronectin were analyzed along the osteogenic protocol. Type 1 collagen is an important protein of the bone extracellular matrix (ECM), where it binds to other proteins and cell surface integrins. Although present in many other cell types, type 1 collagen mediates cell differentiation of the osteoblast phenotype, and its mRNA level generally increases 2 days after osteogenesis induction [46,47]: for this reason, it is considered an early marker of osteo-differentiation. In fact, its expression was evident both in OXT-treated and untreated cells after 3 days from the beginning of the osteogenesis process, and appeared more pronounced in OXT-treated hASCs compared to CTR cells at 7, 14, and 21 days. A similar expression pattern was observed for fibronectin, known to be involved in the early stages of osteogenesis [48]. 

Together these results have shown that OXT promotes osteogenesis and reduces adipogenesis, confirming data already published, albeit in other experimental conditions [32,34].

These findings may be particularly rewarding. In fact, it is increasingly becoming evident that the proneness of suffering a bone fracture is not solely resulting from a decrease in bone mineral density [49]. Rather, preventing the fracturing risk within the context of osteoporosis appears to require the preservation of peculiar cues within the bone ECM. To this end, the maintenance of a proper composition in type I collagen and fibronectin will strongly contribute to fracture resistance beyond mineral bone strength. To this end, it is also worthy of considering the emerging role of fibronectin, an evolutionarily conserved glycoprotein detectable in all tissues, in promoting bone fracture healing, acting as a three-dimensional scaffold immediately after trauma, orchestrating the assembly of multiple ECM components [50]. The currently observed capability of OXT to couple an increase in the osteogenic process with an enhanced expression of key players in ECM-mediated tissue strength may set the basis for deploying the use of OXT in a novel landscape of bone and fracture toughness. The potential biomedical implication of our observations is further supported by the compelling evidence for a remarkable excess of mortality risk in both women and men following an osteoporosis-related hip fracture [51], as well as the persistence of mortality excess in a wide variety of non-hip fragility fractures [52].

## 4. Materials and Methods

### 4.1. hASCs: Harvesting, Culture, and Characterization

hASCs were isolated from the adipose tissue of 3 healthy female subjects, as previously described [36], and this study was approved by the Institutional Ethics Committee Sant’Orsola-Malpighi University Hospital of Bologna, project identification code EM468-2019, reference 6/2016/U/Tess/AOUBo, 22 May 2019. Briefly, hASCs were isolated from the Stromal-Vascular Fraction (SVF) obtained by mechanical mincing and enzymatic digestion of adipose tissue. Samples were digested with Collagenase II 0.075% (Sigma-Aldrich Co., St. Louis, MO, USA) at 37 °C for 30 min in gentle agitation and centrifuged to eliminate oil. Pellets were treated with a hemolytic solution (VersaLyse; Beckman Coulter, Brea, CA, USA) and then centrifuged. The SVFs were plated in 175 cm^2^ flasks (Corning Incorporated, Corning, NY, USA) at the density of 25,000 cells/cm^2^. Cells were cultured in Dulbecco’s Modified Eagle’s Medium—1 g/L of glucose (L-DMEM, Corning Incorporated, Corning, NY, USA) supplemented with 10% Fetal Bovine Serum (FBS; Gibco, Waltham, MA, USA) and antibiotics (1% Penicillin-Streptomycin Solution; Thermo-Fisher Scientific, Waltham, MA, USA) and were maintained in standard culture conditions at 37 °C with 5% carbon dioxide (CO_2_) in a humidified atmosphere. The medium was replaced after 4 days from the seeding and then twice a week until the confluence. When reaching 80% of confluence, hASCs were passaged by treatment with trypsin-EDTA (Sigma-Aldrich Co., St. Louis, MO, USA). Experiments were performed using hASCs at the 3rd–6th culture passage. The hASC characterization and their trilineage differential potential was assessed as described in our previous manuscript [36].

### 4.2. OXT Treatments

OXT was purchased from Sigma-Aldrich (Oxytocin acetate salt hydrate, 1 mg, Cat. Number O6379; Sigma-Aldrich Co., St. Louis, MO, USA) and diluted at 1 mg/mL in sterile H_2_O. Aliquots of OXT solution were stored at −20 °C. For the experiments, OXT was diluted at different concentrations in L-DMEM (range 30–1000 nM). 

### 4.3. Morphological Assessment and OXTR Gene Expression

To analyze hASC morphology, cells were seeded in a 24-well plate (Corning Incorporated, Corning, NY, USA) at a density of 3500 cells/cm^2^. After 24 h in standard conditions, cells were exposed for 72 h to different final concentrations of OXT (100, 500, and 1000 nM). At the end of the exposure time, cells were observed under a light microscope Leica Labovert FS inverted Microscope (Leica Microsystems, Wetzlar, Germany), and images were acquired with a Leica MC170 HD Imaging System Camera (Leica Microsystems, Wetzlar, Germany). 

To evaluate the expression of the *OXTR*, hASCs were seeded in 25 cm^2^ flasks (Corning Incorporated, Corning, NY, USA) at a density of 3500 cells/cm^2^. After 24 h in standard conditions, cells were treated with OXT 1000 nM for 72 h, and then harvested for RNA isolation. Untreated cells were used as CTR. RNA was retrotranscribed in cDNA and used in qPCR analysis (see Section 4.10).

### 4.4. Cell Viability

hASCs were seeded in a 6-well plate (Corning Incorporated, Corning, NY, USA) at a density of 3500 cells/cm^2^. After 24 h in standard conditions, cells were treated with OXT (100, 500, and 1000 nM) for 72 h (2 wells/condition). Then, cells were detached by trypsin-EDTA and counted. Cells were resuspended in a solution containing 50% of Erythrosine B (Sigma-Aldrich Co., St. Louis, MO, USA) red dye 0.2% in Phosphate Buffered Saline (PBS, Corning Incorporated, Corning, NY, USA). Viable and dead cells (not stained and red stained, respectively) were manually counted (at least twice for each condition) using the Neubauer hemocytometer (BRAND GmbH, Wertheim, Germany) and a light microscope Leica Labovert FS inverted Microscope (Leica Microsystems, Wetzlar, Germany). For each sample, cell viability was obtained by calculating the percentage of living cells compared to the total number of cells.

### 4.5. BrdU Assay and Expression Analysis of Proliferation/Cell Cycle Markers

BrdU assay (Roche, Basel, Switzerland) was used to evaluate OXT effects on cell proliferation. hASCs were seeded in a 96-well plate (Corning Incorporated, Corning, NY, USA) at a density of 6000 cells/cm^2^ (3 wells/condition). After 24 h in standard conditions, cells were treated with OXT (100, 500, and 1000 nM) for 72 h and then incubated with 20 μL/well BrdU labeling solution for 3 h at 37 °C, and then fixed with 200 μL/well FixDenat reagent for 30 min at room temperature (RT) (according to the BrdU manufacturer’s instructions). A negative control (medium without cells) was included. Next, hASCs were incubated with peroxidase-conjugated anti-BrdU antibody (AB) and then with the peroxidase substrate. The reaction was interrupted by adding 25 μL of H_2_SO_4_ 1 M to each well. The absorbance at 450 nm was measured with the Wallac 1420 Victor2 Multilabel Counter (Perkin Elmer, Waltham, MA, USA). hASC proliferative ability was expressed as a percentage of BrdU incorporated in treated cells compared to CTR cells (set to 100%) ± standard deviation (SD).

To assess the expression of genes involved in proliferation (*MKI67*) and cell cycle control (*CCND1*, *CDKN2A*, *CDKN1A*, and *TP53*), hASCs were seeded in 25 cm^2^ flasks at a density of 3500 cell/cm^2^. After 24 h in standard conditions, cells were treated with OXT 1000 nM for 72 h, and then harvested for RNA isolation (see Section 4.10). Untreated cells were used as CTR. RNA was retrotranscribed in cDNA and then used in qPCR analysis (see Section 4.10).

### 4.6. Scratch Wound Healing Assay

hASC migration capability was assessed by performing a scratch wound healing assay. hASCs were seeded in a 24-well plate at a density of 20,000 cells/cm^2^ (3 wells/condition). After 24 h in standard conditions, cells were treated with OXT (100, 500, and 1000 nM) for 24 h. Then, a scratch was made using sterile plastic tips. Wells were washed with PBS to remove detached cells. Fresh culture medium with or without OXT was added to the wells. Scratched monolayers were monitored, and images were acquired using a light microscope (Leica Labovert FS inverted Microscope; Wetzlar, Germany) with a Leica MC170 HD digital camera (Leica Microsystems, Wetzlar, Germany) at regular interval times (0, 12, 24, 36 h) until their complete closure. Scratch areas were measured using the ImageJ software 1.53e (National Institute of Health, Bethesda, MD, USA) according to the method described by Venter and Niesler [53]. Briefly, images were converted to 8-bit (grayscale), the edges found (Process *→* Find Edges), and the images blurred (Process *→* Smooth: ×20 times). To correctly identify the wound area, the threshold was manually adjusted. The wound area was quantified by analyzing particles of size between 150 μm to infinity (Analyze *→* Analyze Particles [size: 150—infinity]). The results are presented as the mean of the percentage of wound closure normalized on the CTR ± standard error of the mean (SEM). The percentage of wound closure at t = 0 h was set to 0 for both treated and untreated cells.

### 4.7. Senescence-Associated β-Galactosidase Staining and Gene Expression Analysis of Senescence/Autophagy Markers

Cell staining was performed using a SA β-gal commercial kit (Cell Signaling Technology, Danvers, MA, USA) according to the manufacturer’s instructions. Briefly, hASCs were seeded in a 24-well plate at a density of 3500 cells/cm^2^ (2 wells/condition). After 24 h in standard conditions, cells were treated with OXT (100, 500, and 1000 nM) for 72 h. After the exposure time, cells were fixed and processed according to the manufacturer’s instructions. The staining reaction was incubated overnight. The number of positive (blue) and negative (not colored) cells was counted in each condition in 5 random fields (10 fields/condition) under the microscope Leica Labovert FS inverted Microscope (Leica Microsystems, Wetzlar, Germany). Results are expressed as the percentage of SA β-gal-positive cells ± SD.

To study the expression of genes involved in senescence (*BMI-1* and *TERT*) or in autophagic processes (*BECN1* and *MAP1LC3A*), hASCs were seeded in 25 cm^2^ flasks at a density of 3500 cell/cm^2^. After 24 h in standard conditions, cells were treated with OXT 1000 nM for 72 h, and then harvested for RNA isolation (see Section 4.10). Untreated cells were used as CTR. RNA was retrotranscribed in cDNA and then used in qPCR analysis (see Section 4.10).

### 4.8. Adipogenic Commitment: O.R.O Staining and PPARγ Expression Analysis

The adipogenic differentiation capability of hASCs was evaluated in the presence of OXT for 14 days. Cells were seeded in 48-well or in 6-well plates (Corning Incorporated, Corning, NY, USA) at a density of 9000 cells/cm^2^ (2 wells/condition) for O.R.O staining and gene expression analysis, respectively. At 100% confluence, cells were cultured with standard medium (SM) or StemPro Adipogenesis Differentiation medium (Thermo-Fisher Scientific, Waltham, MA, USA) in the presence or absence of OXT. Untreated cells, both in SM and adipogenic medium, were used as control (CTR). Media were changed twice a week for 14 days.

The qualitative and quantitative assessment of adipogenic differentiation was evaluated in hASCs cultured in the absence or presence of OXT (30, 100, 500, and 1000 nM) with the O.R.O staining solution at the end of the differentiation protocol (14 days). Briefly, cells were fixed with 4% formaldehyde in PBS for 45 min at RT, and then they were stained with a filtered O.R.O solution (Sigma Aldrich Co., St. Louis, MO, USA), 0.2% *w*/*v*, in 60% 2-propanol (VWR International, Radnor, PA, USA) for 30 min. Cells positive for adipogenesis showed red vacuoles in the cytoplasm. Image acquisition for O.R.O staining was performed under phase contrast illumination with the Leica MC170 HD digital camera (Leica Microsystems, Wetzlar, Germany). Five images were acquired for each well. Unstained undifferentiated and stained differentiated cells were counted using ImageJ software [54]. Data are reported as the mean of the percentage of O.R.O positive cells ± SD.

For the expression analysis of the *PPARγ* adipogenic marker, hASCs were treated only with OXT 1000 nM. RNA was extracted and retrotranscribed, as described in Section 4.10, at 3, 7, and 14 days from the beginning of the induction protocol. Finally, the obtained cDNA was used in qPCR analysis (see Section 4.10).

### 4.9. Osteogenic Commitment: Alizarin Red S Staining and Expression Analysis of Osteogenic/Autophagy Markers

Cells were seeded in 24-well plates or 6-well plates at density of 5000 cells/cm^2^ (2 wells/condition) for Alizarin Red S staining and gene expression analysis, respectively. At 70% confluence, cells were cultured with standard medium (SM) or StemPro Osteogenesis Differentiation medium (OM) (Thermo-Fisher Scientific, Waltham, MA, USA) in the presence or absence of OXT. Untreated cells, both in SM and OM, were used as CTR. Media were changed twice a week for 21 days.

The hASC capability to differentiate toward the osteogenic lineage was assessed with a qualitative and quantitative evaluation of calcium deposits in the absence or presence of OXT (30, 100, 500, and 1000 nM) at the end of the differentiation protocol (21 days). Briefly, cells were fixed with 4% formaldehyde in PBS (Sigma-Aldrich Co., St. Louis, MO, USA) in a deionized water solution (pH = 4.1) for 15 min. The exceeding dye was removed by washing wells with deionized water. Images were acquired using the Leica Labovert FS Inverted Microscope (Leica Microsystems, Wetzlar, Germany) with a Leica MC170 HD Imaging System Camera (Leica Microsystems, Wetzlar, Germany). To quantify the number of calcium deposits, the Alizarin Red S dye was solubilized with 10% of acetic acid (Sigma-Aldrich Co., St. Louis, MO, USA); the solution was loaded in a 96-well plate in technical triplicate to measure the absorbance (Abs) at 405 nm using a spectrophotometer plate reader (Wallac 1420 Victor2 Multilabel Counter; Perkin Elmer, Waltham, MA, USA). Data are reported as the mean of the Abs ± SD.

For the expression analysis of *BGLAP*, *BECN1,* and *MAP1LC3A* (osteogenic and autophagy markers, respectively), hASCs were treated only with OXT 1000 nM. RNA was extracted and retrotranscribed, as described in Section 4.10, at 3, 7, 14, and 21 days after the beginning of the induction protocol. Finally, the obtained cDNA was used in qPCR analysis (see Section 4.10).

### 4.10. RNA Extraction, RT-PCR, and qPCR 

For each investigated experimental condition, total RNA was extracted by using the RNeasy Mini Kit (QIAGEN, Valencia, CA, USA) and digested with RNase-free Deoxyribonuclease I (RNase-free DNase set QIAGEN, Valencia, CA, USA) following the manufacturer’s instructions. RNA was then reverse transcribed in cDNA (iScript™ RT Supermix; Bio-Rad Laboratories, Inc., Hercules, CA, USA). To verify whether the retrotranscription reaction was successful, *Glyceraldehyde-3-Phosphate Dehydrogenase* (*GAPDH*) amplification and amplicon detection were performed as previously described in our research [55,56].

For each qPCR analysis, 25 ng of cDNA was amplified using the SsoAdvanced Universal SYBR Green Supermix (Bio-Rad Laboratories, Hercules, CA, USA) in technical triplicates using the Bio-Rad CFX96 real-time thermal cycler (Bio-Rad Laboratories, Hercules, CA, USA), as previously described [36,55,57]. The gene expression was assessed by CFX Manager Software version 3.1 (Bio-Rad Laboratories, Hercules, CA, USA) using the “delta-delta CT method” [58]. For each experimental condition, the expression and the stability of reference genes (*Ribosomal Protein L13a*—*RPL13a*, *GAPDH*, *TATA Binding Protein*—*TBP*, *Hypoxanthine Phosphoribosyltransferase 1*—*HPRT1*) were tested, according to Hellemans and collaborators [59] and the most stable of them were used to normalize the expression of target genes.

*GAPDH*, *TBP*, *HPRT1*, *CDKN2A*, *CDKN1A*, *TP53*, *BMI-1*, and *TERT* primers were purchased from Bio-Rad (20X, Bio-Rad Laboratories, Hercules, CA, USA); all the other sequences were provided from Sigma-Aldrich (Sigma-Aldrich Co., St. Louis, MO, USA). The list of primer sequences is reported in Table 2. For each gene, the normalized expression value of CTR cells was set to 1, and all other gene expression values were reported to that value. Data are expressed as normalized fold change ± SEM.

### 4.11. Immunofluorescence Analysis of Osteogenic Markers

hASCs were seeded in 24-well plate containing glass coverslips (Corning Incorporated, Corning, NY, USA) at density of 5000 cells/cm^2^ (2 wells/condition). At 70% confluence, cells were cultured with StemPro OM in the presence or absence of OXT 1000 nM. Untreated cells were used as CTR. For the evaluation of RUNX2, OPN, type I collagen, and fibronectin protein expression, cells were fixed at 3, 7, 14, and 21 days starting from the beginning of the induction protocol, respectively. Cells were fixed with 4% formaldehyde (Sigma-Aldrich Co., St. Louis, MO, USA) for 15 min and then washed with PBS-Tween 0.25% (Sigma-Aldrich Co., St. Louis, MO, USA). Cells were permeabilized with Triton X-100 0.25% and sodium citrate 10 mM (Sigma-Aldrich Co., St. Louis, MO, USA) in PBS for 15 min at RT. Samples were blocked for 1 h with a solution containing 4% Bovine Serum Albumin (BSA; Sigma-Aldrich Co., St. Louis, MO, USA) and 0.3% Triton X-100 in PBS. To evaluate the RUNX2 protein expression and visualize matrix deposition, cells were incubated for 3 h at RT with the following primary ABs: rabbit anti-Runx2 (AB23981; Abcam, Cambridge, UK), mouse anti-OPN (AB69498; Cambridge, UK), mouse anti-type I collagen (AB6308, Abcam, Cambridge, UK), and mouse anti-fibronectin (SC-8422, Santa Crutz Biotechnology, Dallas, TX, USA). Then, cells were incubated with the appropriate fluorescence-conjugated secondary ABs (anti-rabbit Fluorescein Isothiocyanate or anti-mouse conjugated with Tetramethylrhodamine-5-(and 6)-isothiocyanate) for 1 h, at RT. Primary and secondary ABs were diluted in a solution containing 2% BSA (Sigma-Aldrich Co., St. Louis, MO, USA) and 0.15% Triton X-100 in PBS (dilution 1:200). Incubation with NucBlue^®^ Fixed Cell Ready-Probes^®^ Reagent (DAPI, Molecular Probes™, Life Technologies—Thermo-Fisher Scientific, Waltham, MA, USA) was used to counterstain nuclei. All slides were mounted with the antifade AF-400 (Immunological Sciences, Rome, Italy). The detection and acquisition of images were performed using Nikon Inverted Microscope Eclipse Ti2-E (Nikon Instruments, Melville, NY, USA) and a Digital Sight camera DS-Qi2 (Nikon Instruments, Melville, NY, USA) through the imaging software NIS-Elements. The fluorescence intensity was quantified by using Fiji ImageJ software 2.1.0/1.53c [60] as described by Vidoni et al., 2019 [44]. Fluorescence intensity was indicated as IntDen (Integrated Density), and it refers to the average value of fluorescence in a selected area normalized to the number of cells. Fluorescence was quantified in 20 microscopic fields randomly chosen. Data are presented as the mean of IntDen ± SEM.

### 4.12. Statistical Analysis

Data were analyzed by using GraphPad Prism software 5.03 (GraphPad Software, San Diego, CA, USA) and qBase software 2.3 (Biogazelle qbasePLUS). Data were tested for normality, following which appropriate parametric tests (One-Way ANOVA and post hoc Tukey Test) or nonparametric equivalents (Kruskal–Wallis’s test with post hoc Dunn’s test) were used. Results are shown as mean ± SD or mean ± SEM. A *p*-value < 0.05 was considered statistically significant.

## 5. Conclusions

In recent years, the importance of hASCs in the promising field of cell therapy has increasingly emerged, in particular for their differentiative capacity, such as that towards osteogenic commitment. In the present study, we have shown that OXT, which we already know plays an important role in health and well-being, is able to promote hASCs towards an osteogenic commitment, acting as a driving force in the early days of the differentiation pathway. In association with this, we observed how OXT is also able to activate autophagy marker genes in the same period of time. The capability of OXT to elicit a significant increase in type I collagen and fibronectin, together with an increase in osteogenesis and autophagy, may set the basis for novel bone and fracture toughness approaches beyond those devoted mainly to promoting bone strength via an increase in bone mineral density. Further in vitro and in vivo studies will be needed to address this wide area of inquiry.

## Figures and Tables

**Table 1:** Viability of human adipose-derived stem cells (hASCs) after 72 h of Oxytocin (OXT) treatment.

	72 h
CTR	96.9 ± 2.2
OXT 100 nM	95.5 ± 1.4
OXT 500 nM	95.3 ± 1.6
OXT 1000 nM	95.4 ± 1.8

72 h: time of OXT-treatment; CTR: control hASCs; OXT 100 nM, OXT 500 nM, and OXT 1000 nM: hASCs treated with OXT at concentrations of 100, 500, and 1000 nM, respectively. Data are expressed as the mean percentage of living cells ± standard deviation (SD); *n* = 3.

**Table 2:** Primer sequences of the genes analyzed by qPCR in hASCs.

Gene	Entrez Gene ID *	Left Primer	Right Primer	Bio-Rad Unique Assay ID	A.L. (bp) ^$^
*Glyceraldehyde 3-phosphate dehydrogenase* *(GAPDH)*	2597	-	-	qHsaCED0038674	117
*TATA box binding protein* (*TBP)*	6908	-	-	qHsaCID0007122	120
*Hypoxanthine phosphoribosyl transferase 1* *(HPRT1)*	3251	-	-	qHsaCID0016375	90
*Ribosomal Protein L13a* *(RPL13a)*	23521	TGAAGGAGTACCGCTCCAAAC	GGAGACTAGCGAAGGCTTTGA	-	233
*Oxytocin Receptor* *(OXTR)*	5021	TCTTCTTCGTGCAGATGTGG	GGACGAGTTGCTCTTTTTGC	-	236
*Proliferation marker protein Ki-67* *(MKI67)*	4288	TCAGACTCCATGTGCCTGAG	TTGTCCTCAGCCTTCTTTGG	-	134
*Cyclin-dependent kinase inhibitor 2A* *(CDKN2A or p16^INK4a^)*	1029	-	-	qHsaCED0056722	86
*Cyclin-dependent kinase inhibitor 1A* *(CDKN1A or p21)*	1026	-	-	qHsaCID0014498	159
*Tumor protein p53* *(TP53)*	7157	-	-	qHsaCID0013658	126
*Cyclin D1* *(CCND1)*	595	CAGATCATCCGCAAACACGC	AAGTTGTTGGGGCTCCTCAG	-	143
*BMI1 proto-oncogene, polycomb ring finger* *(BMI-1)*	648	-	-	qHsaCED0046537	78
*Telomerase reverse transcriptase* (*TERT)*	7015	-	-	qHsaCID0009247	150
*Beclin1* *(BECN1)*	8678	AACCAGATGCGTTATGCCCA	TCCATTCCACGGGAACACTG	-	148
*Microtubule-associated protein 1 light chain 3 alpha* *(MAP1LC3A)*	84557	TTGGTCAAGATCATCCGGCG	CCTGGGAGGCGTAGACCATA	-	163
*RUNX family transcription factor 2* *(RUNX2)*	860	CTCCCTGAACTCTGCACCAA	TAGAGTGGATGGACGGGGAC	-	149
*Peroxisome proliferator-activated receptor gamma* *(PPARγ)*	5468	TTGCAGTGGGGATGTCTCAT	TTTCCTGTCAAGATCGCCCT	-	208
*Bone gamma-carboxyglutamic acid-containing protein* *(BGALP)*	632	CACCGAGACACCATGAGAGC	CTGCTTGGACACAAAGGCT	-	132

* ID: identification number; ^$^ A.L. (bp): Amplicon length (base pair).

## Data Availability

The datasets used and/or analyzed during the current study are available from the corresponding author upon reasonable request.
